# Coronavirus disease-2019: is fever an adequate screening for the returning travelers?

**DOI:** 10.1186/s41182-020-00201-2

**Published:** 2020-03-09

**Authors:** George M. Bwire, Linda S. Paulo

**Affiliations:** 1 School of Pharmacy, Muhimbili University of Health and Allied Sciences, P.O. Box 65001, Dar es Salaam, Tanzania; 2 School of Public Health and Social Sciences, Muhimbili University of Health and Allied Sciences, P.O. Box 65001, Dar es Salaam, Tanzania

**Keywords:** COVID-19, Fever, Returning travelers, Temperature screening

## Abstract

On Thursday, 30 January 2020, World Health Organization declared Coronavirus disease-2019 (COVID-2019) a Public Health Emergency of International Concern. Since its identification in late December 2019 in Wuhan, Hubei Province, People’s Republic of China, the number of cases imported into other countries is increasing, and the epidemiological map is changing rapidly. On the other hand, body temperature screening (fever) is the major test performed at points of entry, i.e., airports, in the returning travelers in most of the countries with limited resources. However, the recent report on asymptomatic contact transmission of COVID-19 and travelers who passed the symptoms-based screening and tested positive for COVID-19 using reverse transcription polymerase chain reaction (RT-PCR) challenges this approach as body temperature screening may miss travelers incubating the disease or travelers concealing fever during travel. On this note, travel restrictions to and from high risk areas and/or 14 days quarantine of travelers coming from high risk areas are recommended to prevent possible importation of COVID-19. Currently, RT-PCR is a reliable test in detecting both symptomatic and asymptomatic COVID-19.

To the Editor,

Since the outbreak of Coronavirus on December 31, 2019 in Wuhan, Hubei Province, People’s Republic of China, the number of cases from China that have been imported into other countries is increasing, and the epidemiological map is changing rapidly [1]. In this regard, World Health Organization (WHO) on Thursday, 30 January 2020, announced a Coronavirus disease-2019 (COVID-19) a Public Health Emergency of International Concern (PHEIC).

To curb the spread of COVID-19, WHO has implemented several public health measures including rapid identification, diagnosis and management of the cases, identification and follow-up of contacts, infection prevention and control in health care settings, implementation of health measures for travelers, awareness rising in the population, and risk communication [2].

Additionally, countries are taking measures to prevent the importation of COVID-19, i.e., on January 31, 2020, the President of the USA signed a presidential “Proclamation on Suspension of Entry as Immigrants and Non-immigrants of Persons who Pose a Risk of Transmitting COVID-19” [3]. Other measures implemented elsewhere include quarantine of travelers coming from risk countries and symptoms-based screening at points of entry [4], which were also employed during Ebola outbreaks. In support of this approach, WHO reported that temperature screening at entry detected the majority of exported cases during the current outbreak with the COVID-19 [5].

However, 4 people in Germany were infected with Coronavirus-2019 through contact with an asymptomatic patient [6], although there is a claim that the patient from China who transmitted the virus when she/he was attending the business meeting in Germany was symptomatic [7]. But, evidence is still clear that the other 2 patients who never came into contact with the Chinese patient contracted the virus from an asymptomatic German patient who attended the meeting with a Chinese patient. This added to the ongoing debates about virus transmission, where some of the scientists believe that asymptomatic patients are transmitting the infection. WHO says “People who cough or sneeze are more likely to spread the virus,…But even if they do, asymptomatic transmission likely plays a minor role in the epidemic overall.” Additionally, evidence from Germany reported that 2 out of 114 travelers (1.8%) from Wuhan, China, who had passed the symptoms-based screening tested positive for COVID-19 by reverse transcription polymerase chain reaction (RT-PCR) [8].

More interestingly, Japanese citizens evacuated from the source of a COVID-19 outbreak that have been diagnosed with the infection after initially testing negative, a man in his 50s who returned from the Chinese city of Wuhan on the first Japanese evacuation flight on January 29, 2020, previously twice tested negative for the virus. However, a third test 12 days later found the man—who has been isolated in his hotel room since his return was infected. In addition to that, the number of people in Diamond Princess testing positive for COVID-19 keep on increasing, since it arrived off Japan [9].

A case from the UK reports on the British businessman who is linked to 11 coronavirus cases that a suspect was advised to attend an isolated room at hospital, despite showing no symptoms and later tested positive for COVID-19 [10]. National Health Service has advised any individual experiencing symptoms, even if mild, after traveling from mainland China, Thailand, Japan, Republic of Korea, Hong Kong, Taiwan, Singapore, Malaysia, or Macau, to stay indoors. In Africa, Egypt is the first country to confirm COVID-19 infection and the country’s health ministry says the affected person is a non-Egypt citizen (foreigner) who presented with no serious symptoms [11]. The second COVID-19 case in Africa has been reported to occur in Algeria from a foreigner coming from the northern part of Italy who arrived in the country on Feb 17, 2020. Northern Italy, home to many Algerians, has been the center of an outbreak of the coronavirus with more than 280 cases and 11 deaths [12]. From Latin America, Brazil is the first country to confirm the case of COVID-19 in a 61-year-old who had visited Italy [13].

Lastly, a published study on clinical characteristics of 138 hospitalized patients with COVID-19 in Wuhan, China, documented that fever was present in 98.6% (136/138) of hospitalized patients, whereas 2 non-intensive care unit patients (1.4%) did not present with fever [14]. In this regard, body temperature might not be an adequate screening as it can potentially miss travelers incubating the disease or travelers concealing fever during travel and contribute to the importation of the virus to the countries of destination. Therefore, travel restrictions to and from high risk areas [1] and/or 14-day quarantine [15] of people coming from high risk areas are recommended to prevent possible importation of COVID-19. Currently, RT-PCR is a reliable test in detecting both symptomatic and asymptomatic COVID-19 [6, 8]. Lastly, in previous experience from other viral outbreaks, i.e., dengue virus [16] and Ebola [17], fever screening especially at airport had a positive effect on partially blocking importation of cases. Kuan et al. [16] reported that airport fever screening was successful in identifying 45% (244/542; 95% confidence interval 33.1–57.8%) of imported dengue cases with fever.

## Data Availability

Data used to draw the conclusion are provided in this letter. Conclusions drawn in this letter reflect authors’ views and not the Institution of Affiliation.

## Abbreviations

COVID-19: Corona virus disease-2019
PHEIC: Public Health Emergency of International Concern
RT-PCR: Reverse transcription polymerase chain reaction
WHO: World Health Organization
